# Amorphization Optimization of Ge_2_Sb_2_Te_5_ Media for Electrical Probe Memory Applications

**DOI:** 10.3390/nano8060368

**Published:** 2018-05-25

**Authors:** Lei Wang, Cihui Yang, Jing Wen, Bangshu Xiong

**Affiliations:** School of Information Engineering, Nanchang Hang Kong University, Nanchang 330069, China; yangcihui@nchu.edu.cn (C.Y.); wenj@nchu.edu.cn (J.W.); 42021@nchu.edu.cn (B.X.)

**Keywords:** Ge_2_Sb_2_Te_5_, amorphization, probe, optimization, model

## Abstract

Electrical probe memory using Ge_2_Sb_2_Te_5_ media has been considered a promising candidate in the future archival storage market due to its potential for ultra-high density and long data retention time. However, most current research efforts have been devoted to the writing of crystalline bits using electrical probe memory while ignoring the viability of writing amorphous bits. Therefore, this paper proposes a physical, realistic, full three-dimensional model to optimize the practicable media stack by spatially and temporally calculating temperature distributions inside the active media during the writing of amorphous bits. It demonstrates the feasibility of using an optimized device that follows a Silicon/Titanium Nitride/Ge_2_Sb_2_Te_5_/Diamond-Like Carbon design with appropriate electro-thermal properties and thickness to achieve ultra-high density, low energy consumption, and a high data rate without inducing excessive temperature. The ability to realize multi-bit recording and rewritability using the designed device is also proven, making it attractive and suitable for practicable applications.

## 1. Introduction

**Figure 1:**
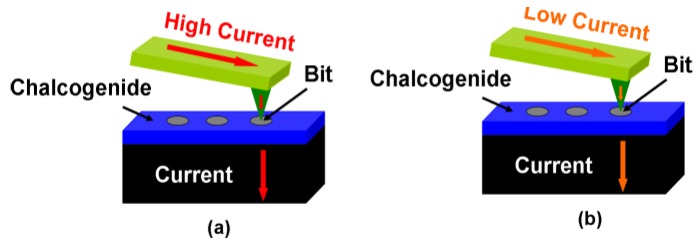
The electrical probe memory using Chalcogenide media when operated in (**a**) write mode and (**b**) readout mode.

Data storage devices play a crucial role in citizens’ daily life as a result of the proliferation of computerized data triggered by the global digitalization storm. Accordingly, global digital data has increased at an unprecedented rate, much higher than that of conventional data storage devices such as the magnetic hard disk, magnetic tape, and optical disc. Under such circumstances, research has focused on the development of innovative memory devices that can satisfy the current storage requirements from either industry or consumers. Recently, electrical probe memory has been considered as one of the most promising storage devices for the future archival storage application due to its superior recording and replay performances when compared to its predecessors [1]. Electrical probe memory usually comprises a conductive probe tip that serves as the top electrode, a storage stack that consists of a Chalcogenide alloy (e.g., Ge_2_Sb_2_Te_5_ media) surrounded by a protective capping layer and a bottom electrode, both of which are deposited into the silicon (Si) wafer. The recording process of electrical probe memory is achieved by raising the temperature inside the Chalcogenide layer through Joule heating, by means of an appropriate electric stimulus applied either to the crystallization temperature to induce the crystalline mark embedded inside the amorphous background or to the melting temperature, followed by fast cooling and quenching of the molten materials to generate amorphous marks surrounded by crystalline background. The replay process is accomplished by applying a low readout voltage through the conductive tip to the storage stack and, subsequently, sensing the readout current variations caused by the large resistance contrast between crystalline/amorphous marks and their respective background. These variations can be further adjusted by reversing the bias voltages which are linked to the solid-state electrolytic behavior [2]. The principles of the recording and replay processes of the electrical probe memory are schematically shown in Figure 1.

The advantageous features of the electrical probe memory derive from the use of the nanoscale probe tip, leading to the possibility of securing ultra-high recording density; additionally, the Chalcogenide alloy acts as storage media to provide super-fast phase transition, long data retention, low energy consumption, excellent scalability, and high On/Off resistance contrast [3,4]. Given its performance superiorities over conventional storage devices, the electrical probe memory has recently received considerable attentions from worldwide researchers in the aspects of the experimental fabrication [2,3,4,5,6,7] and the theoretical simulation [8,9,10]. It should be noted that the majority of previous research concentrated on the phase transition from amorphous to crystalline phases while ignoring the writing of amorphous marks from the crystalline background. Undoubtedly, the writing of crystalline marks from an amorphous background typically costs less energy than its counterpart and thus exempts high temperatures from appearing inside the capping layer. However, this writing mechanism severely deteriorates its rewritability, as erasing the previously written crystalline mark partially crystallizes the neighbouring amorphous region. Consequently, this results in a peculiar crystalline ‘halo’ which encompasses the erased region, thereby degrading both the readout signal and the achievable recording density [8]. In this case, the recording of the electrical probe memory based on the crystalline-to-amorphous transformation (i.e., amorphization) is more desirable than the amorphous-to-crystalline transition (i.e., crystallization) in terms of its rewritability and, accordingly, worth particular investigation. Notably, the GeTe-Sb_2_Te_3_-based superlattice material has recently become a hot spot as a result of its potentially lower switching energy and faster switching speed [11,12]. However, as a result of increased knowledge regarding the electro-thermal and mechanical properties of the Ge_2_Sb_2_Te_5_ media since its debut for storage applications, the active media of the electrical probe memory studied here is focused on the Ge_2_Sb_2_Te_5_ layer. 

## 2. Modeling

Prior to the study of the amorphization of the electrical probe memory, a physical-realistic model is required to provide insight into temporally and spatially resolved kinetics of writing current, device temperature, and phase transition for the required amorphized process. As amorphization closely pertains to temperature and cooling rate inside the storage layer, the designed model, similar to the previous crystallization case [9], includes several equations, comprising the Laplace equation and the heat transfer equation to compute temperature and cooling rate inside the Chalcogenide layer (i.e., Ge_2_Sb_2_Te_5_ in this paper), given by

(1) ∇⋅(σ⋅∇V)=0,

(2) $$\rho C_{p}\frac{\partial T}{\partial t}-k\cdot \nabla^{2}T=\sigma {\left|E\right|}^{2},$$

where *σ* is the electrical conductivity; *V* is the electric potential; *ρ* is the density; *C_p_* is the specific heat; *T* is the temperature; *k* is the thermal conductivity; *E* is the electric field.

**Figure 2:**
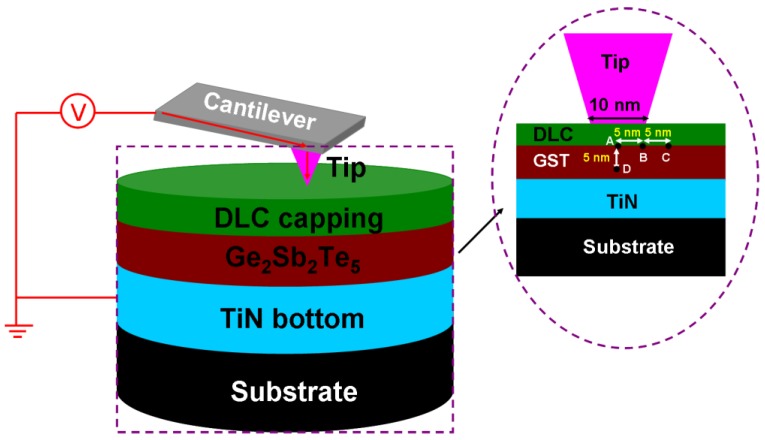
The 3D modeling geometry of the electric probe memory and its cross section (inset) where GST represents Ge_2_Sb_2_Te_5_.

The Laplace equation is solved to provide the electric field and current density distributions inside the Ge_2_Sb_2_Te_5_ layer, which are simultaneously implemented as the heat source for the heat transfer equation, performing the calculations of temperature and cooling rate distributions inside the Ge_2_Sb_2_Te_5_ layer. In our simulation, the crystalline Ge_2_Sb_2_Te_5_ turned into its amorphous state in the region where the temperature exceeded the melting point (i.e., 620 °C) [8], and the cooling rate was greater than 37 °C/ns [8]. In this case, the electrical conductivity of the Ge_2_Sb_2_Te_5_ alloy was defined by:

(3) $$\sigma_{\mathrm{GST}}=\sigma_{\mathrm{am}}\cdot (T>=620\;{}^{\circ}C)\cdot (T_{t}\geq 37\;{}^{\circ}C/\mathrm{ns})+\sigma_{\mathrm{cryst}}\cdot ((T<620\;{}^{\circ}C)\|\left(T_{t}<37\;{}^{\circ}C/\mathrm{ns}\right)),$$

where *T_t_* is the cooling rate, and *σ*_am_ and *σ*_cryst_ are the electrical conductivity of the Ge_2_Sb_2_Te_5_ alloy in its amorphous and crystalline phases respectively, given by [6]:

(4) $$\sigma_{\mathrm{am}}=\sigma_{0\mathrm{am}}\times \exp(-\Delta \xi_{\mathrm{am}}/k_{B}T)\times \exp(E/E_{0}),$$

(5) $$\sigma_{\mathrm{cryst}}=\sigma_{0\mathrm{cryst}}\times \exp(-\Delta \xi_{\mathrm{cryst}}/k_{B}T),$$

where *σ*_0am_ and *σ*_0cryst_ are prefactors for amorphization and crystallization with a value of 1.88 × 10^4^ Ω^−1^·m^−1^ and 1.5 × 10^4^ Ω^−1^·m^−^^1^, respectively; Δ*ξ*_am_ and Δ*ξ*_cryst_ are the activation energies for the temperature dependence of amorphous *σ*_GST_ and crystalline *σ*_GST_ with a value of 0.32 eV and 0.04 eV, respectively; *E*_0_ is the critical electric field with a value of 5 × 10^7^ V·m^−1^ and k_B_ is the Boltzmann Constant. The thermal conductivity of the Ge_2_Sb_2_Te_5_ alloy is described in a similar manner to its electrical conductivity, given by:

(6) $$k_{\mathrm{GST}}=k_{\mathrm{am}}\cdot (T>=620\;{}^{\circ}C)\cdot (T_{t}<=-37\;{}^{\circ}C/\mathrm{ns})+k_{\mathrm{cryst}}\cdot ((T<620\;{}^{\circ}C)\|\left(T_{t}>-37\;{}^{\circ}C/\mathrm{ns}\right)),$$

where *k*_am_ and *k*_cryst_ are the thermal conductivities of amorphous and crystalline Ge_2_Sb_2_Te_5_ with a value of 0.2 W·m^−1^·K^−1^ and 0.58 W·m^−1^·K^−1^, respectively. To more closely mimic the practical setup, all simulations in this paper were performed in fully three-dimensional (3D) environments. Previous literature has reported that amorphization of the Ge_2_Sb_2_Te_5_ media can be induced using the conductive probe by directly contacting the probe tip with the phase-change layer [2]. However, if the phase-change layer were exposed directly to the air, it would be easily prone to oxidation and wear and, therefore, is not practicable for the real design. In this case, it was important to overcoat phase-change layer with a capping layer which met the electrical requirements for successful writing and reading while providing oxidation protection and wear resistance. Accordingly, a typical electrical probe memory consists of a conductive probe and a trilayer stack, which is made up of a Ge_2_Sb_2_Te_5_ layer sandwiched by a diamond-like carbon (DLC) layer and a titanium nitride (TiN) bottom electrode, as illustrated in Figure 2. Such an architecture has been previously used to optimize the writing of crystalline bits [9,10]. A commercial software package based on the finite-element method, i.e., Comsol Multiphysics^TM^, was deployed to imitate the electrical, thermal, and phase-transformation kinetics which occurred inside the device. The write or readout pulse was applied to the conductive probe while the bottom TiN electrode was grounded. These two boundaries were also maintained at room temperature, whereas all others were assumed to be electrically and thermally insulated. The values of characteristic parameters used in the model are given in Table 1.

## 3. Results

Several design requirements needed to be fulfilled to successfully optimize the amorphization without incurring any adverse effects on device. To achieve multi-Terabits/in^2^ density, a small diameter of the probe tip was required, as it determined the conductive region through which current could flow, and thus strongly affect the bit size [13]. In our simulation, the diameter of the tip was restricted to 10 nm to provide ~10 Tbit/in^2^, which corresponded to a bit diameter of ~10 nm. Such writing should also be accomplished without inducing excessive temperature inside the device (predominantly the thin DLC capping layer); the resulting thermal cross-talk that can overwrite the adjacent written bit must be strongly suppressed. These requirements have been used to define a set of maximum and minimum temperatures that should be realized at various locations in the storage media during the amorphization process.

To write amorphous bits with ~10 Tbit/in^2^, the temperatures in the Ge_2_Sb_2_Te_5_ layer at points A (at the top of the Ge_2_Sb_2_Te_5_ layer and under the tip), B (at the top of the Ge_2_Sb_2_Te_5_ layer and at the tip edge being 5 nm away from A), and D (in the middle of the Ge_2_Sb_2_Te_5_ layer, being 5 nm away from A) needed to reach a minimum of 620 °C so as to satisfy one of the two indispensable conditions for amorphization. Additionally, because the maximum temperature of the device took place approximately at A, the temperature at A was required to be less than 1000 °C to maintain the thermal stability of the DLC capping [14]. Finally, the temperature at point C, where the edge of the adjacent written bit was located, could not exceed 200 °C so as not to weaken the resulting thermal cross-talk [15]. Therefore, it was necessary to optimize the prototype of the electrical probe memory shown in Figure 1 so as to simultaneously meet the aforementioned maximum and minimum temperature requirements. 

**Figure 3:**
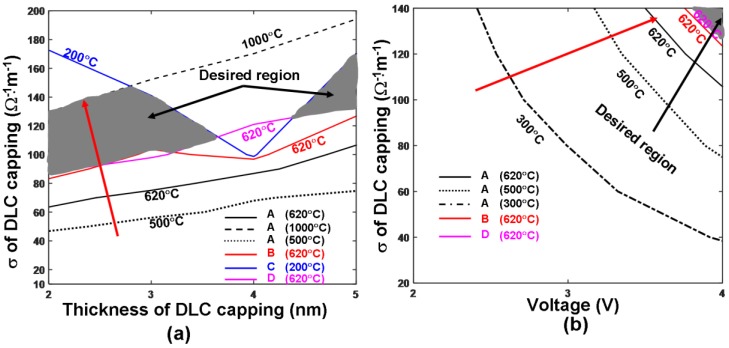
Temperature contours at points A, B, C, D as a function of (**a**) electrical conductivities and thickness of the DLC capping layer; and (**b**) electrical conductivities and tip voltages. For both simulations, the thermal conductivities of the capping and under layer remained at 0.5 W·m^−1^·K^−1^ and 12 W·m^−1^·K^−1^, while other modeling parameters are given in Table 1. Note that for (**b**) maximum temperature contours of 1000 °C at A and 200 °C at C are outside this figure and, therefore, are not visible. The red arrow indicates the direction along which the temperature at A increased.

The electrical conductivity and the thickness of the DLC capping layer have previously been found to play a critical role in determining the crystallization extent of the Ge_2_Sb_2_Te_5_ layer, as it serves as a conductive bridge for write current to flow into the active media. The optimum values of these parameters for the writing of crystalline bits fall within the range of 10 Ω^−1^·m^−1^ to 100 Ω^−1^·m^−1^ (electrical conductivity), and 2 nm to 4 nm (thickness) [9]. Therefore, the minimum and maximum temperatures at locations A, B, C, and D, were calculated by varying the electrical conductivity and the thickness of the DLC capping from 10 Ω^−1^·m^−1^ to 200 Ω^−1^·m^−1^, and from 2 nm to 5 nm to cover the range of possible values reported previously [9,16]. The tip voltage was set at a 4 V pulse of 120 ns, with 100 ns rising and 20 ns trailing edge (such a voltage was chosen to induce sufficiently high temperature for amorphization while achieving the appropriate cooling rate), resulting in Figure 3a. As can be seen from Figure 3a, the temperature inside the Ge_2_Sb_2_Te_5_ layer continuously increased by either reducing the capping layer thickness or enhancing the electrical conductivity of the DLC capping. This was expected, as both strategies could lower the whole device resistance and thus induce more Joule heating inside the Ge_2_Sb_2_Te_5_ layer for a given pulse. However, notably, a thin capping layer with a very high electrical conductivity was not anticipated, as this would have caused excessively high temperatures in the capping layer (>1000 °C) and the adjacent written bit (>200 °C), thereby adversely affecting the device performances. In this case, the optimum values for the electrical conductivity and the thickness of the DLC capping that can fulfill the aforementioned maximum and minimum temperature requirements were mainly concentrated on the two gray regions highlighted in Figure 3a. As indicated in Figure 3a, the optimum values for electrical conductivity and thickness of DLC capping within the left gray region ranged from 100 Ω^−1^·m^−1^ to 150 Ω^−1^·m^−1^, and from 2 nm to 3.5 nm. In contrast, for the right gray region, these optimum parameters varied from 140 Ω^−1^·m^−1^ to 160 Ω^−1^·m^−1^, and from 4.5 nm to 5 nm. Undoubtedly, to design a practicable device, the values of these characteristic parameters are pertinent to the inherent properties of materials which suggest that a thicker DLC layer usually exhibits a higher electrical conductivity [17]. Additionally, recent literature has reported that the electrical conductivity of a 5 nm DLC capping layer, when subjected to Joule heating, can sharply increase to 140 Ω^−1^·m^−1^ [18,19]; in contrast, a DLC capping layer with an electrical conductivity of 140 Ω^−1^·m^−1^ and a thickness of 5 nm appears to be a satisfying configuration that can simultaneously meet the temperature requirements for amorphization as well as the practicable measurements.

Apart from the target for ultra-high density, the write pulse for amorphization must be carefully determined so that the write power and energy of a real device can be minimized. To investigate this, the minimum and maximum temperatures at locations A, B, C, and D were recalculated by both varying the electrical conductivities of the DLC capping from 20 Ω^−1^·m^−1^ to 140 Ω^−1^·m^−1^ to cover the previously designed optimum values and adjusting the pulse magnitudes from 2 V to 4 V, as depicted in Figure 3b. Notably, the 200 °C and 1000 °C contours were outside the plot (i.e., they only occurred for voltage beyond 4 V). Clearly, the write pulses of either 2 V or 3 V were incapable of generating the required amorphization temperature within the electrical conductivity range investigated in Figure 3b; the above temperature requirements could only be satisfied by implementing a write pulse of 4 V and a DLC capping with an electrical conductivity between 130 Ω^−1^·m^−1^ and 140 Ω^−1^·m^−1^. As a result, the use of a 4 V pulse of 120 ns and a DLC capping with an electrical conductivity of 140 Ω^−1^·m^−1^ and a thickness of 5 nm is an optimum choice for writing amorphous bits inside the electrical probe memory.

**Figure 4:**
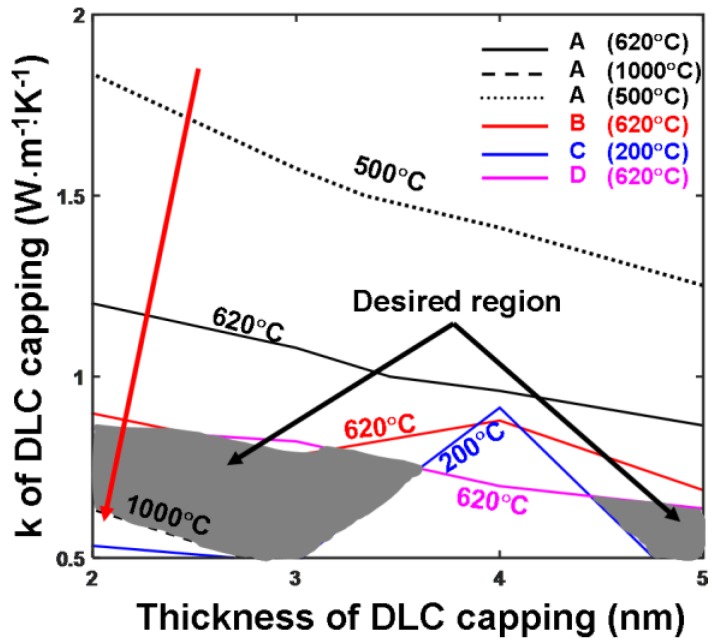
Temperature contours at points A, B, C, D as a function of thermal conductivities and thickness of the DLC capping layer. For both simulations, the electrical conductivities of the capping and under layer remained at 0.5 W·m^−1^·K^−1^ and 12 W·m^−1^·K^−1^, while other modeling parameters are given in Table 1. The write pulses for all simulations were maintained as 4 V of 120 ns. The red arrow indicates the direction along which the temperature at A increased.

In addition to electrical conductivity, thermal conductivity of the DLC capping was previously found to play a critical role in determining the temperature distribution inside the Ge_2_Sb_2_Te_5_ layer during its crystallization process [9]. Accordingly, we assessed the influence of the thermal conductivity of the DLC capping on the aforementioned maximum and minimum temperature requirements by changing its thermal conductivity and thickness from 0.5 W·m^−1^·K^−1^ and 2 nm to 2 W·m^−1^·K^−1^ and 5 nm from to cover the possible reported values [20,21], leading to Figure 4. The results shown in Figure 4 clearly suggest that increasing the thermal conductivity of DLC capping reduces the temperature in the detected locations for a given write pulse, as this reinforces the heat spreading effect, and inversely prevents the original transformed region from acquiring sufficient heat. As a result, a higher write pulse is required to achieve essential temperatures for amorphization when using a DLC capping with a large thermal conductivity. According to Figure 4, the optimum values of the thermal conductivities also lie in the two gray regions. The left region exhibits a thermal conductivity from 0.5 W·m^−1^·K^−1^ to 0.8 W·m^−1^·K^−1^ and a thickness from 2 nm to 3.5 nm, whereas the thermal conductivity and the thickness inside the right region ranges from 0.5 W·m^−1^·K^−1^ to 0.75 W·m^−1^·K^−1^ and from 4.5 nm to 5 nm. To match above optimization findings for electronic parameters of the DLC capping (i.e., 140 Ω^−1^·m^−1^ and 5 nm) as well as practicable measurements, 0.5 W·m^−1^·K^−1^ was selected as the optimized thermal conductivity of the DLC capping.

**Figure 5:**
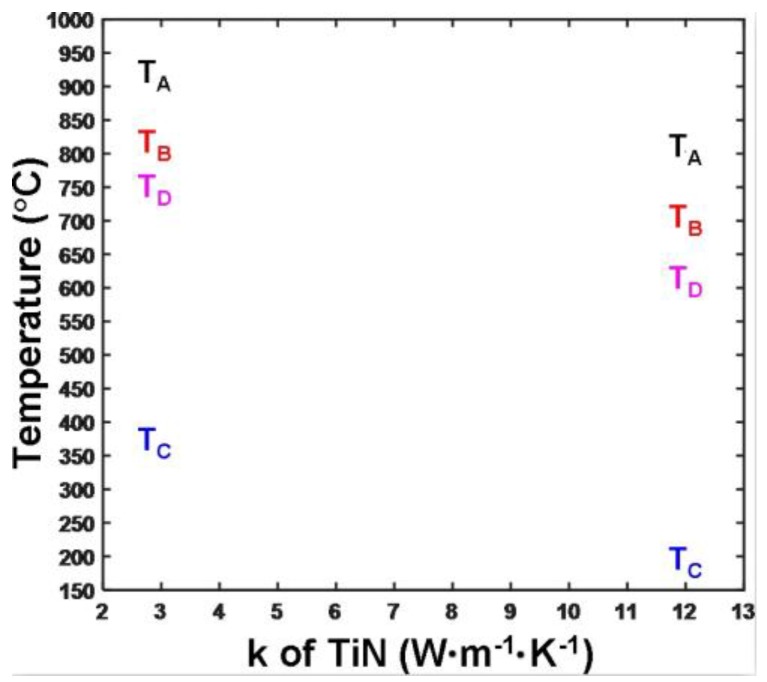
Calculated temperature at points A, B, C, D as a function of thermal conductivities of the TiN bottom electrode (i.e., 3 W·m^−1^·K^−1^ and 12 W·m^−1^·K^−1^). During simulations, the thickness, electrical conductivity, and thermal conductivity of the capping layer were set to be 5 nm, 140 Ω^−1^·m^−1^, and 0.5 W·m^−1^·K^−1^, respectively, while other modeling parameters are given in Table 1. The write pulses for all simulations were maintained as 4 V of 120 ns.

Notably, the electro-thermal properties of the TiN bottom layer also needed to be carefully determined, as they predominantly function as a bottom electrode where the write current is collected and, accordingly, may affect the resulting Joule heating. To investigate this, the minimum and maximum temperatures at aforementioned locations were recomputed by altering the electrical conductivity of the TiN layer from 1 × 10^6^ Ω^−1^·m^−1^ to 1 × 10^7^ Ω^−1^·m^−1^, with a thermal conductivity of either 3 W·m^−1^·K^−1^ or 12 W·m^−1^·K^−1^ to include the experimentally reported values [22], as illustrated in Figure 5. Notably, for the TiN layer with a thermal conductivity of 12 W·m^−1^·K^−1^, Tc was below 200 °C, and is therefore not visible in Figure 5. On the one hand, it was determined that the electrical conductivity of the TiN bottom electrode had a negligible impact on the resulting temperatures at these points (and, accordingly, not shown in Figure 5), which arose from the fact that the electrical conductivity of the TiN layer was much higher than those of the DLC capping and the Ge_2_Sb_2_Te_5_ layer. As a result, any variation in its electrical conductivity rarely affected the whole device resistance and the write current. On the other hand, using a TiN bottom layer with lower thermal conductivity (3 W·m^−1^·K^−1^) induced higher temperatures at detected locations than the larger thermal conductivity case (12 W·m^−1^·K^−1^). This was expected, because the bottom electrode with larger thermal conductivity allowed Joule heat to flow more readily towards the Si substrate which acted as a heat sink as opposed to staying inside the active media, thereby resulting in a lower temperature inside these locations. Further observations in Figure 5 reveal that the temperature requirements for points A, B, and D could be satisfied by the TiN layer with either 3 W·m^−1^·K^−1^ or 12 W·m^−1^·K^−1^, whereas the 3 W·m^−1^·K^−1^ case exhibited a temperature of 350 °C at point C that clearly exceeded the defined safety range. For this reason, the electrical conductivity and the thermal conductivity of the TiN bottom electrode were chosen to be 1 × 10^7^ Ω^−1^·m^−1^ and 12 W·m^−1^·K^−1^_,_ respectively.

## 4. Discussion

**Figure 6:**
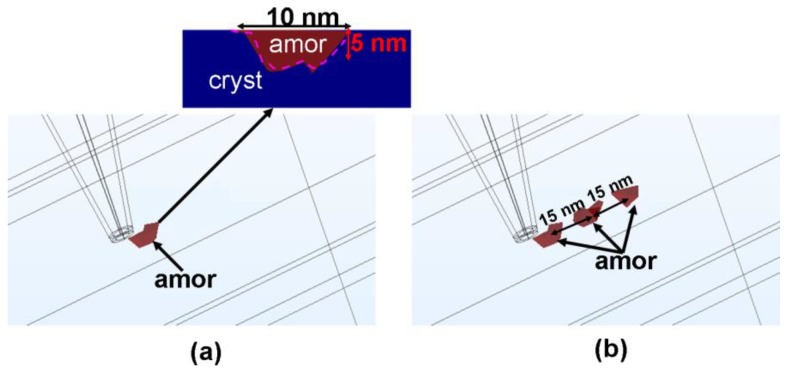
The writing of an isolated amorphous bit (**a**) and multiple amorphous bits (**b**) based on the optimized device architecture and a 4 V pulse of 120 ns. The inset shows the cross-section image of the written isolated amorphous bit bounded by the purple dash.

According to the results, to optimize the writing of amorphous bits in the crystalline staring phase, a design of an electrical probe memory structure is proposed here that comprises the following (as seen in Figure 6): a conductive probe with a diameter of ~10 nm as well as a storage media stack consisting of a 10 nm Ge_2_Sb_2_Te_5_ layer sandwiched by a 5 nm DLC capping layer with electrical and thermal conductivities of 140 Ω^−1^·m^−1^ and 0.5 W·m^−1^·K^−1^, respectively, in addition to a 40 nm TiN layer with electrical and thermal conductivities of 1 × 10^7^ Ω^−1^·m^−1^ and 12 W·m^−1^·K^−1^, respectively. As can be seen from Figure 6a, integrating the aforementioned optimized device design with a 4 V pulse of 120 ns enables a formation of an amorphous bit with a diameter of 10 nm, corresponding to multi-Terabits/in^2^ recording density. Notably, as illustrated in the inset of Figure 6a, the written amorphous bit did not have to extend through the whole thickness of the active media which contrasts with the writing of crystalline bits in which it is preferred to extend through the whole thickness. This is because the resistance of the amorphous bits is much higher than its surrounding crystalline background; as a result, the readout current induced when the tip is on top of the amorphous bit is still much lower than the case with the tip on top of the crystalline surrounding, thus easily distinguishing the recording bit from its background. Accordingly, it is not necessary to amorphize the entire region underneath the tip, which allows for lower writing energy and results in a writing energy per bit of ~2 pJ that suggests for 0.2 nJ and 2 nJ for using 100 and 1000 tips in parallel. Furthermore, the use of pulse duration of 120 ns readily enables a data rate per tip of 1 Mbit/s, which can be even dramatically boomed for parallel probe recording.

**Figure 7:**
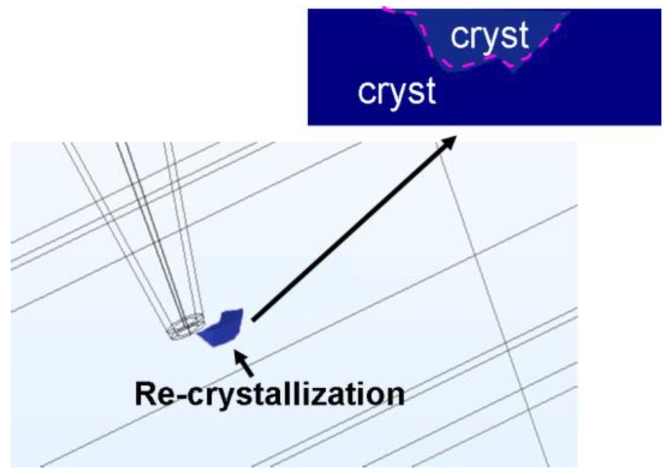
The erasing (re-crystallization) of the previously written amorphous bit in Figure 6a by a 4 V pulse of 100 ns. The inset shows the cross-section image of the re-crystallized bit bounded by the purple dash.

To more closely model the real environment, the main functions of the electrical probe device, such as multi-bit recording and rewritability, which functioned as the adopted scenarios in practice, needed to be reflected in the developed 3D model. To simulate the writing of multiple bits, the conductive probe was first brought to the position where amorphization was expected to occur; the write pulse was subsequently applied to the conductive probe to perform the recording, after which the probe was moved to the next data point. It was found in Figure 6b that three consecutive amorphous bits, with a 10 nm diameter and a center-to-center distance of 15 nm, were formed by means of the above optimized device architecture, further demonstrating its attractive potential for ultra-high density. Additionally, observations that the contiguous region between two adjacent amorphous bits remained crystalline indicated an effective inhibition of the resulting thermal cross-talk phenomenon which frequently arises during the writing of crystalline bits. This result ensured the reliability of the device. In addition to multi-bit recording, the erasure of a previously written amorphous bit (i.e., recrystallization) was also simulated, leading to Figure 7. Clearly, such an erasing process involves crystallization kinetics which can be accurately modelled by simultaneously solving a set of coupled equations, including the Laplace equation, the heat conduction equation, and the crystallization rate equation, all of which have been previously proposed for the optimization in the writing of crystalline bits [10] and, consequently, have not been repeated here. According to Figure 7, by choosing the appropriate erasing pulse (4 V of 100 ns here), the previously written amorphous bit could be completely recrystallized without adversely re-amorphizing the surrounding crystalline background (i.e., the temperature inside the surrounding crystalline region did not exceed the melting point). This simulation result suggests that re-writeable probe storage should be feasible by first writing amorphous bits into a crystalline staring phase, followed by erasure by re-crystallization.

## 5. Conclusions

A full 3D theoretical model that involves the electrical, thermal, and amorphization processes of the Ge_2_Sb_2_Te_5_ media for probe storage applications was developed to optimize the architecture of the electrical probe memory by spatially and temporally calculating temperature distributions inside the active media during the writing of amorphous bits. The feasibility of using the optimized device that follows a Si/TiN/Ge_2_Sb_2_Te_5_/DLC design with appropriate electro-thermal properties and thickness to achieve ultra-high density, low energy consumption, and high data rate without inducing excessive temperature in the device was demonstrated. The ability to realize multi-bit recording and rewritability using the designed device as also proven, making it attractive and suitable for practicable applications.

## Figures and Tables

**Table 1:** Characteristic parameters used in the model.

Parameter	TipDLC	DLC	GST	TiN	Si
Thinckness (nm)	10	2–5	10	40	1000
*ρ* (Kg·m^−3^)	12,400	2800	6150	5400	2330
*C_p_* (J·Kg^−1^·m^−3^)	250	540	210	400	720
*K* (W·m^−1^·K^−1^)	25	0.5–2	Equation (3)	3 or 12	149
*σ* (Ω^−1^·m^−^^1^)	3.3 × 10^−^^6^	10–200	Equation (4)	1 × 10^6^–1 × 10^7^	N/A

## References

[1] Wang L., Yang C., Wen J., Peng Y.X. (2016). Overview of probe-based storage technologies. Nanoscale Res. Lett..

[2] Pandian R., Kooi B.J., Palasantzas G., De Hosson J.T.M., Pauza A. (2007). Nanoscale electrolytic switching of in phase-change chalcogenide films. Adv. Mater..

[3] Sun X., Rob U., Gerlach J.W., Lotnyk A., Rauschenbach B. (2017). Nanoscale bipolar switching of Ge_2_Sb_2_Te_5_ phase-change material thin films. Adv. Electron. Mater..

[4] Wong H.S.P., Raoux S., Kim S., Liang J., Reifenberg J.P., Rajendran B., Asheghi M., Feng S., Goodson K. (2010). Phase-change memories. Proc. IEEE.

[5] Gidon S., Lemonnier O., Rolland B., Bichet O., Dressler C. (2004). Electrical probe storage using joule heating in phase change media. Appl. Phys. Lett..

[6] Satoh H., Sugawara K., Tanaka K. (2006). Nanoscale phase changes in crystalline Ge_2_Sb_2_Te_5_ films using scanning probe microscopes. J. Appl. Phys..

[7] Bhaskaran H., Sebastian A., Pauza A., Pozidis H., Despont M. (2009). Nanoscale phase transformation in Ge_2_Sb_2_Te_5_ using encapsulated scanning probes and retraction force microscopy. Rev. Sci. Instrum..

[8] Wright C.D., Marilyn M., Aziz M.M. (2006). Terabit-per-square-inch data storage using phase-change media and scanning electrical nanoprobes. IEEE Trans. Nanotechnol..

[9] Wang L., Wen J., Yang C., Gai S., Peng Y. (2015). The route for ultra-high recording density using probe-based data storage device. Nano.

[10] Wang L., Gong S.D., Wen J., Yang C.H. (2016). An improved electrical switching and phase-transition model for scanning probe phase-change memory. J. Nanomater..

[11] Simpson R.E., Fons P., Kolobov A.V., Fukaya T., Krbal M., Yagi T., Tominaga J. (2011). Interfacial phase-change memory. Nat. Nanotechnol..

[12] Lotnyk A., Hilmi I., Ross U., Rauschenbach B. (2018). Van der waals interfacial bonding and intermixing in GeTe-Sb_2_Te_3_-based superlattices. Nano Res..

[13] Bhaskaran H., Sebastian A., Drechsler U., Despont M. (2009). Encapsulated tip for reliable nanoscale conduction in scanning probe technologies. Nanotechnology.

[14] Kalish R., Lifshitz Y., Nugent K., Prawer S. (1999). Thermal stability and relaxation in diamond-like carbon. A Raman study of films with different sp^3^ fractions (ta-C to a-C). Appl. Phys. Lett..

[15] Wuttig M., Yamada N. (2007). Phase-change materials for rewritable data storage. Nat. Mater..

[16] Robertson J. (2002). Diamond like amorphous carbon. Mater. Sci. Eng. R.

[17] Wang L., Gong S.D., Yang C.H., Wen J. (2016). Towards low energy consumption data storage era using phase-change probe memory with TiN bottom electrode. Nanotechnol. Rev..

[18] Bachmann T.A., Alexeev A.M., Koelmans W.W., Zipoli F., Ott A.K., Dou C., Ferrari A.C., Nagareddy V.K., Craciun M.F., Jonnalagadda V.P. (2017). Temperature evolution in nanoscale carbon-based memory devices due to local Joule heating. IEEE Trans. Nanotechnol..

[19] Santini C.A., Sebastian A., Marchiori C., Jonnalagadda V.P., Dellmann L., Koelmans W.W., Rossell M.D., Rossel C.P., Eletheriou E. (2015). Oxygenated amorphous carbon for resistive memory applications. Nat. Commun..

[20] Balandin A.A., Shamsa M., Liu W.L., Casiraghi C., Ferrari A.C. (2009). Thermal conductivity of ultrathin tetrahedral amorphous carbon films. Appl. Phys. Lett..

[21] Shamsa M., Liu W.L., Balandin A.A., Casiraghi C., Milne W.I., Ferrari A.C. (2006). Thermal conductivity of diamond-like carbon film. Appl. Phys. Lett..

[22] Giraud V., Cluzel J., Sousa V., Jacquot A., Dauscher A., Lenoir B., Scherrer H., Romer S. (2005). Thermal characterization and analysis of phase change random access memory. J. Appl. Phys..

